# Levocarnitine regulates the growth of angiotensin II-induced myocardial fibrosis cells via TIMP-1

**DOI:** 10.1515/biol-2022-0554

**Published:** 2023-02-09

**Authors:** Jin Shu, Jue Shi, Yiwen Gu, Lei Deng, Chen Zhao, Chun Wu, Jiachen Zhao, Haiya Wang, Li Jin

**Affiliations:** Department of Gerontology, Shibei Hospital of Jing’an District, Shanghai, 200443, China; Department of Gerontology, Shanghai Ninth People’s Hospital, Shanghai Jiaotong University School of Medicine, Shanghai, 200023, China

**Keywords:** tissue inhibitor of metalloproteinases-1, myocardial fibrosis, levocarnitine, angiotensin II

## Abstract

This study aimed to explore the effects of tissue inhibitor of metalloproteinases‐1 (TIMP‐1) on levocarnitine (LC)-mediated regulation of angiotensin II (AngII)-induced myocardial fibrosis (MF) and its underlying mechanisms. H9C2 cells were treated with AngII for 24 h to induce fibrosis. The cells were then treated with LC or transfected with TIMP‐1-OE plasmid/si‑TIMP‐1. Cell apoptosis, viability, migration, and related gene expression were analyzed. AngII treatment significantly upregulated *Axl*, *α-SMA*, and *MMP3* expression (*P* < 0.05) and downregulated *STAT4* and *TIMP1* expression (*P* < 0.05) relative to the control levels. After transfection, cells with TIMP-1 overexpression/knockdown were successfully established. Compared with that of the control, AngII significantly inhibited cell viability and cell migration while promoting cell apoptosis (*P* < 0.05). LC and TIMP-1-OE transfection further suppressed cell viability and migration induced by Ang II and upregulated apoptosis, whereas si-TIMP-1 had the opposite effect. Furthermore, LC and TIMP-1-OE transfection downregulated *Axl, AT1R, α-SMA, collagen III, Bcl-2*, and *MMP3* expression caused by AngII and upregulated caspase 3, p53, and *STAT4* expression, whereas si-TIMP-1 had the opposite effect. TIMP-1 is therefore a potential therapeutic target for delaying MF progression.

## 1 Introduction

Heart failure (HF) is a global threat affecting 23 million people worldwide, and its prevalence continues to increase over time [1]. At a certain stage, myocardial fibrosis (MF) may be the leading cause of vasomotor dysfunction and myocardial disarray in various cardiovascular diseases, characterized by myocardial structural destruction, excessive cardiac interstitial fibroblast proliferation, and deposition [2,3]. Among patients with coronary artery disease, MF is strongly associated with sudden cardiac death [4] and long-term mortality in patients with HF [5]; thus, inhibiting MF progression is highly important. However, current therapeutics, including β-blockers, endothelin antagonists, ATI receptor antagonists, angiotensin-converting enzymes, and statins, usually display significant adverse effects [6,7]. Therefore, there is an urgent need to further explore the pathogenesis of MF and identify novel therapeutic targets to delay MF and improve HF outcomes.

Levocarnitine (LC) has good therapeutic potential for treating cardiovascular disease (HF and myocardial infarction), and a large body of experimental and clinical evidence supports the role of LC in suppressing fibrosis progression [8,9]. LC can improve angiotensin II (AngII)-induced reactive oxygen species (ROS) generation, AT1R expression, and collagen accumulation, thereby inhibiting cardiac fibrosis [10]. Zhao et al. showed that LC could inhibit the expression of proinflammatory and profibrotic cytokines and decrease intracellular production of ROS, followed by a significant reduction in renal tubulointerstitial inflammation and fibrosis [11]. Another study reported that LC protected against cardiac and renal damage by inhibiting oxidative stress and increasing antioxidant enzyme function by inhibiting TNF-α and IL-1β in isoprenaline-treated MI rat models [12]. These reports indicate that LC may affect fibrosis and protect cardiac function; however, the mechanisms of LC in MF progression remain unclear.

Tissue inhibitors of matrix metalloproteinases-1 (TIMP-1), a member of the TIMP family, are glycoproteins expressed in a variety of tissues and organs that can inhibit the activity of matrix metalloproteinase (MMP3) [13]. The role of TIMP-1 in the development of liver fibrosis has been widely reported [14]. Further evidence demonstrated that AngII could reduce TIMP-1 expression via AT1R and phosphokinase C, ultimately affecting collagen deposition [15]. TIMP family members are involved in MF regulation through the alteration of the intercellular matrix via inhibition of the activity of MMP family members [13]. MMP3 is significantly upregulated in AngII-induced MF cells, and its knockdown could delay MF progression by altering the activity of AngII‑induced MF by regulating genes related to oxidative stress and cell apoptosis [16]. These results indicated the importance of TIMP-1 in MF. However, the specific effects of TIMP-1 on the regulation of MF progression by LC remain unclear.

AngII is a cytokine with many biological functions, including growth factor-like effects. By mainly binding to its receptor AT1, AngII can promote the occurrence of MF because it can not only induce cardiomyocyte hypertrophy but also stimulate the proliferation of cardiac fibroblasts and collagen synthesis [17,18]. In the progression of MF, AngII acts as a critical signaling molecule associated with the renin-angiotensin system (RAS) [19]. Cellular changes within fibrosis induced by AngII are similar to those in humans [20]. A previous study by Chen et al. used 100 nM AngII to induce human cardiac fibroblasts to simulate fibrosis for 48 h, and the significant increase in fibrosis-related indicators after Ang II induction suggested the occurrence of fibrosis [21]. Another study applied 100 nM Ang II to stimulate cardiac fibroblasts for 24 h to induce cell fibrosis [22]. These reports demonstrated that AngII-induced MF in H9C2 cells can be used for experiments. Therefore, in this study, AngII was used to treat H9C2 cells for 24 h to induce cellular fibrosis. Next, the cells were treated with LC or transfected with TIMP‐1-OE/si‑TIMP‐1 plasmid to further investigate the roles of TIMP-1 in LC regulation of AngII-induced MF and their related mechanisms. These findings would help to improve our understanding of MF-based cardiovascular diseases and provide new therapeutic strategies for the prevention and delay of MF.

## 2 Materials and methods

### 2.1 Cell culture

H9C2 rat myocardial cells were purchased from the Chinese Academy of Sciences Cell Bank (Shanghai, China). The cells were cultured in Dulbecco’s modified Eagle’s medium (Wuhan Boster Biological Technology) supplemented with 10% fetal bovine serum (FBS) and 1% penicillin/streptomycin (Thermo Fisher Scientific) and then maintained at 37℃ with 5% CO_2_.

### 2.2 Cell transfection

H9C2 cells at a density of 5 × 10^5^ cells per well were seeded into six-well plates and cultured overnight. Then, the complete culture medium was replaced with serum-free medium, and H9C2 cells were transfected with 100 nM si-NC/si-TIMP-1 (1/2/3) or NC/TIMP-1-OE plasmid using Lipofectamine^®^ 3000 (Thermo Fisher Scientific, Inc.), according to the manufacturer’s protocols. NC and TIMP-1-OE plasmids and si-NC/si-TIMP-1 (1/2/3) were purchased from Yanzai Biotechnology (Shanghai) Co., Ltd (Shanghai, China). After 6 h of transfection, the serum-free medium was replaced with a complete culture medium. After culturing for another 24 h, total RNA was isolated from the cells with different treatments, and cell transfection efficiency was assessed by determining TIMP-1 expression using quantitative reverse transcription-polymerase chain reaction (qRT-PCR) and western blotting.

### 2.3 Induction of cellular MF and cell grouping

H9C2 cells (5 × 10^5^ cells per well) were seeded in 12‑well plates and cultured overnight. The next day, cells were treated with 10^−8^ M AngII for 24 h. After 24 h of AngII treatment, the cells were harvested to extract total RNA for the examination of MF- and fibroblast-related genes.

H9C2 cells were divided into five groups: control, MF, MF + LC, MF + LC + si-TIMP-1, and MF + LC + TIMP-1-OE. The cells in the MF and MF + LC groups were first treated with AngII for 24 h and then treated with equal amounts of PBS and 100 µM LC for another 24 h. The cells in the MF + LC + si-TIMP-1 and MF + LC + TIMP-1-OE groups were transfected with si-TIMP-1 and TIMP-1-OE plasmids, respectively. After transfection, TIMP-1 knockdown and overexpression cells were stimulated with 10^−8^ M AngII for 24 h and then treated with 100 µM LC. The cells in the control group were not treated. Cells subjected to different treatments were harvested for subsequent experiments.

### 2.4 Cell viability and apoptosis assays

The Cell Counting Kit-8 (CCK-8) was used to evaluate the effects of TIMP-1 on the viability of fibrotic H9C2 cells. Briefly, cells in the different groups were collected and seeded into a 96-well plate (1 × 10^4^ cells per well). After culturing for 24, 48, 72, and 96 h, 10 µL of CCK-8 reagent was added, and the cells were further incubated for 2.5 h at 37℃. Finally, the absorbance at 450 nm was measured using a microplate reader.

The Annexin V-FITC Apoptosis Detection kit (Beyotime Institute of Biotechnology) was used to assess cell apoptosis, following the manufacturer’s recommendations. Briefly, H9C2 cells with different treatments for 48 h were harvested by centrifugation for 5 min at 1,000×*g* and resuspended in 195 µL of Annexin V-FITC. Then, 10 µL of PI and 5 µL of Annexin V-FITC were added to the cells, and the cells were incubated in the dark for 20 min at 20℃. Finally, a FACSCalibur flow cytometer (Becton-Dickinson) was used to acquire images of cells, and CellQuest software (version 4, Becton, Dickinson and Company) was used to calculate the apoptosis rate (early plus late apoptosis) in the different groups.

### 2.5 Cell migration evaluation

The effects of TIMP-1 on cell migration were investigated using transwell chambers (TCS003024, Guangzhou Jet Bio-Filtration Co., Ltd). Briefly, H9C2 cells with different treatments at a density of 6 × 10^4^ cells per well were seeded into the upper chamber of the transwell chambers. In the lower chamber, the complete medium containing 10% FBS was added. After incubation at 37°C for 24 h, the cells were treated with 4% paraformaldehyde for 10 min and stained with 0.5% crystal violet for 10 min. Finally, a microscope was used to observe cell migration, and the cell numbers were analyzed.

### 2.6 qRT-PCR

Total RNA was isolated from the cells with different treatments using TRIzol^®^ reagent (Thermo Fisher Scientific, Inc.), and the concentration and quality of the isolated RNA were measured using a UV spectrophotometer. Then, using the PrimeScript™ II 1st Strand cDNA Synthesis kit (Takara Bio, Inc.), RNA was reverse-transcribed into cDNA according to the manufacturer’s instructions. Next, SYBR Premix EX Taq (Thermo Fisher Scientific, Inc.) was used to perform qPCR using the ViiA7 real-time PCR system (ABI). The amplification procedure was as follows: 95°C for 3 min and 40 cycles of 95°C for 10 s, 60°C for 30 s, and 60°C for 30 s. mRNA expression levels were calculated using the 2^–ΔΔCt^ method. The primer sequences are listed in Table 1. GAPDH was used as the housekeeping gene.

**Table 1:** Sequences of all primers

Primer	Sequence (5′–3′)
GAPDH	F: AGACAGCCGCATCTTCTTGT
	R: CTTGCCGTGGGTAGAGTCAT
MMP3	F: CAGGCATTGGCACAAAGGTG
	R: GTGGGTCACTTTCCCTGCAT
Axl	F: GCCCAGTGAGTGAACCCC
	R: TCTCCTTCAGCTCTTCGCTG
Bcl-2	F: GACTGAGTACCTGAACCGGCATC
	R: CTGAGCAGCGTCTTCAGAGACA
Caspase 3	F: GAATCCACGAGCAGAGTC
	R: TCAACAAGCCAACCAAGT
p53	F: ACAGTTAGGGGGTACCTGGC
	R: GCTGTGGTGGGCAGAATATCAT
α-SMA-	F: GGAGATGGCGTGACTCACAA
	R: CGCTCAGCAGTAGTCACGAA
TIMP-1	F: TAAAGCCTGTAGCTGTGCCC
	R: AGCGTCGAATCCTTTGAGCA
Collagen III (COL3A1)	F: CGGAGGAATGGGTGGCTATC
	R: ACCAGCTGGGCCTTTGATAC
AT1R	F: GGAAACAGCTTGGTGGTGAT
	R: CACACTGGCGTAGAGGTTGA
STAT4	F: ACCTGAGTAAGGCTGTCC
	R: TCAGTTCCTACCCACTCTAT
COL1A1	F: ACTTAACATCCAAGGCCGCT
	R: ACAATATTTGCCTCAGTTTGTGC
FSP1	F: TGTAATAGTGTCCACCTTCC
	R: TTCATTGTCCCTGTTGCT
TGF-β1	F: ATGGTGGACCGCAACAAC
	R: TGAGCACTGAAGCGAAAGC

### 2.7 Western blotting

The total protein of the cells with different treatments was extracted using RIPA lysis buffer, and a BCA protein assay kit was used to measure the total protein concentrations. After that, the protein samples (20 μg) were separated by 10% SDS-PAGE and transferred onto polyvinylidene difluoride membranes. After blocking with 5% skim milk at 37°C for 2 h, the membranes were incubated with anti-Axl antibody (1:1,000; Protein Tech Group, Inc.), anti-α-SMA antibody (1:1,000; Protein Tech Group, Inc.), anti-MMP3 antibody (1:1,000; Protein Tech Group, Inc.), anti-STAT4 antibody (1:1,000; Protein Tech Group, Inc.), anti-TIMP1 antibody (1:200; Santa Cruz, Inc.), anti-p53 antibody (1:2,000; Protein Tech Group, Inc.), anti- Caspase3 antibody (1:1,000; Protein Tech Group, Inc.), and anti-GAPDH antibody (1:10,000; Protein Tech Group, Inc.) at 4°C overnight. On the next day, the secondary antibody (goat anti-mouse IgG (H + L)-HRP [1:10,000; Jackson ImmunoResearch Laboratories, Inc.]) was added, and cells were incubated at 37°C for 2 h. Finally, the membrane was visualized using an ECL assay kit (Beyotime Institute of Biotechnology), and the protein expression levels of the related proteins were quantified using Image-Pro Plus software.

### 2.8 Statistical analysis

Data are shown as mean ± standard deviation (SD). GraphPad Prism software was used for statistical analysis. The homogeneity of variance test was first performed to analyze the homogeneity of the variance of the data. According to the *P*-value of the homogeneity of variance test, one-way ANOVA followed by Tukey’s *post hoc* test was performed if *P* > 0.05; otherwise, Brown-Forsythe and Welch ANOVA tests followed by Dunnett’s T3 *post hoc* test were performed. Differences were considered statistically significant at *P* < 0.05.

## 3 Results

### 3.1 Expression of MF markers in AngII-induced cells and cell transfection efficiency

**Figure 1:**
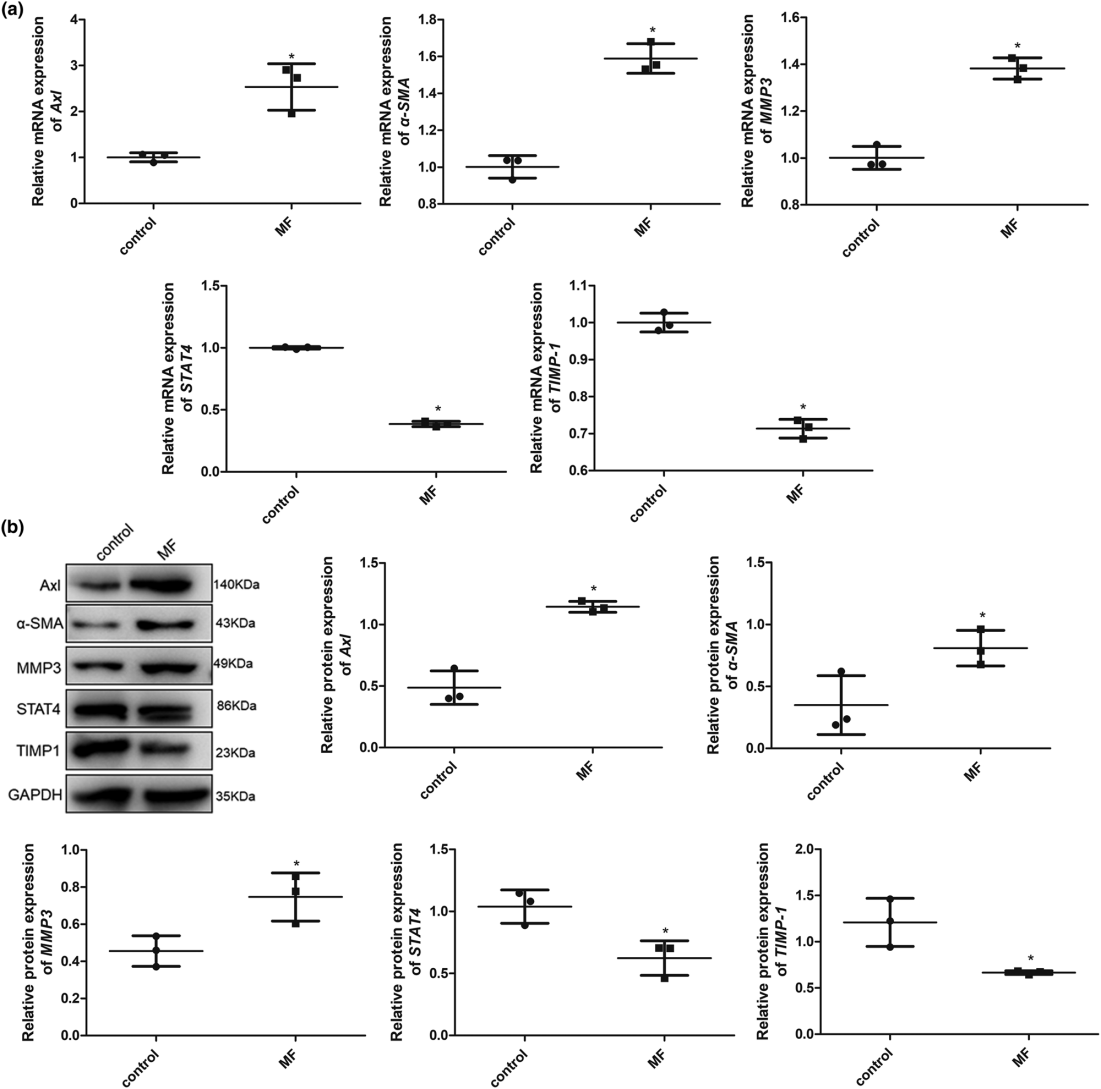
Expression of MF-related markers in AngII-induced cells using qRT-PCR and western blotting. (a) mRNA expression of *Axl*, *α-SMA*, *MMP3*, *STAT4*, and *TIMP-1* after AngII treatment for 24 h determined using qRT-PCR. (b) Protein expression of Axl, α-SMA, MMP3, STAT4, and TIMP-1 after AngII treatment for 24 h determined using western blotting. *P < 0.05 vs control. MF, myocardial fibrosis.

**Figure 2:**
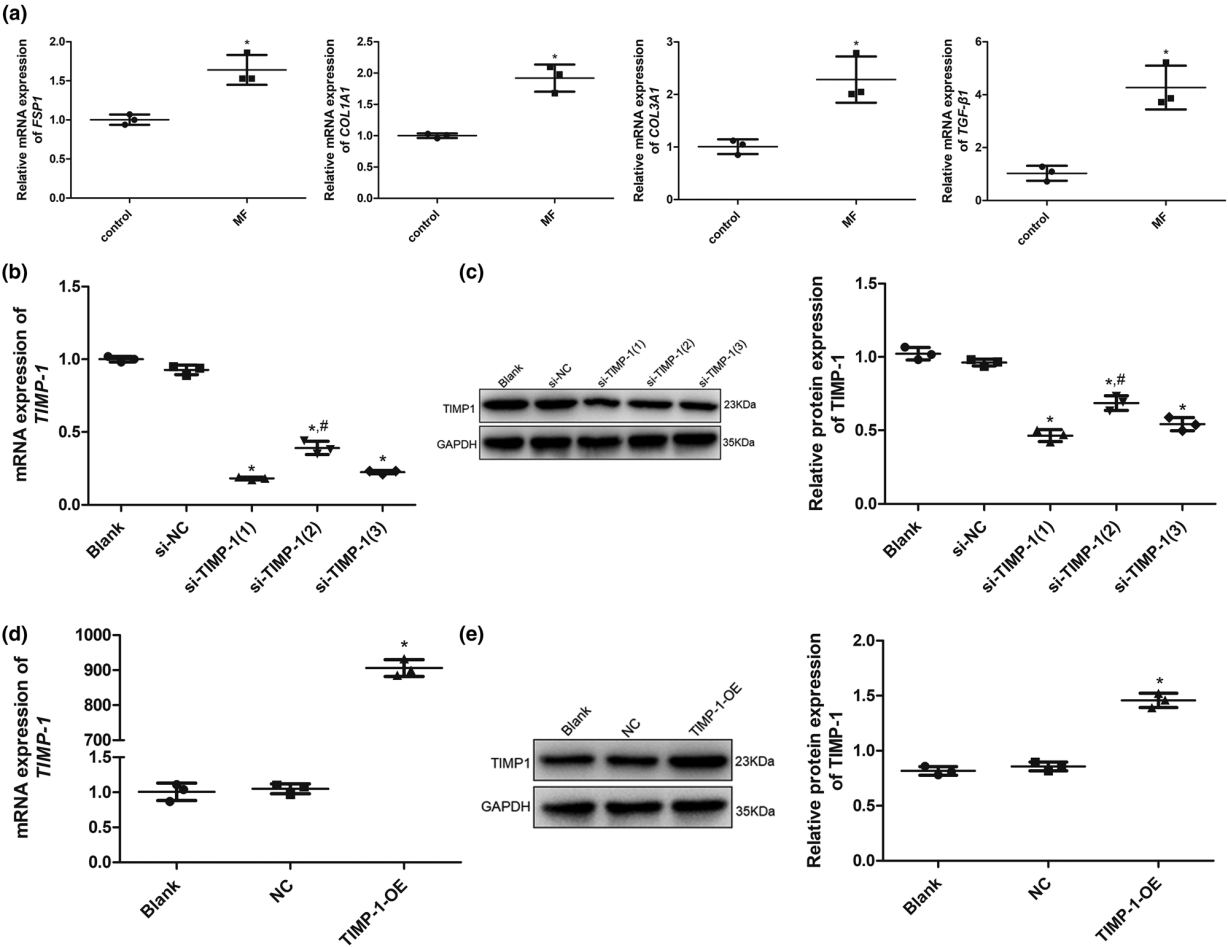
Expression of fibroblast-related markers in AngII-induced cells and cell transfection efficiency. (a) mRNA expression of *FSP1*, *COL1A1*, *COL3A1*, and *TGF-β1* after AngII treatment for 24 h determined using qRT-PCR. *P < 0.05 vs control. (b) mRNA expression of *TIMP-1* after transfection with si-TIMP-1. (c) Protein expression of *TIMP-1* after transfection with si-TIMP-1. (d) mRNA expression of *TIMP-1* after transfection with TIMP-1-OE plasmid. (e) Protein expression of *TIMP-1* after transfection with TIMP-1-OE plasmid. *P < 0.05 vs blank, ^#^ *P* < 0.05 vs si-TIMP-1(1). TIMP‐1, tissue inhibitor of metalloproteinases‐1; MF, myocardial fibrosis; si, small interfering RNA; OE, over-expression.

As shown in Figure 1a, after Ang II treatment for 24 h, the mRNA expression of *Axl*, *α-SMA*, and *MMP3* was significantly upregulated (*P* < 0.05), while that of *STAT4* and *TIMP1* was downregulated (*P* < 0.05) compared with the control cells. Western blotting also showed that the trends of Axl, α-SMA, MMP3, STAT4, and TIMP-1 protein expression in the control and MF groups were similar to that of their mRNA expression (Figure 1b). Additionally, the expression of fibroblast-related markers, such as *FSP1*, *COL1A1*, *COL3A1*, and *TGF-β1*, was determined using qRT-PCR. The mRNA expression levels of *FSP1*, *COL1A1*, *COL3A1*, and *TGF-β1* were significantly higher in the MF group than in the control group (*P* < 0.05, Figure 2a). These results indicate that AngII successfully induced the formation of fibrotic H9C2 cells.

To investigate the roles of TIMP-1 in LC regulation of MF, H9C2 cells were transfected with TIMP-1-OE plasmid and si-TIMP-1, and cell transfection efficiency was evaluated by measuring TIMP-1 expression using qRT-PCR and western blotting. There was no significant difference in *TIMP-1* mRNA and protein expression between the blank and si-NC groups (*P* > 0.05, Figure 2b and c) or between the blank and NC groups (*P* > 0.05, Figure 2d and e). Compared with that in the blank group cells, the mRNA and protein expression of *TIMP-1* in the cells transfected with si-TIMP-1(1), si-TIMP-1(2), and si-TIMP-1(3) was significantly decreased (*P* < 0.05). si-TIMP-1(1) had the most significant effect (Figure 2b and c); hence, H9C2 cells with TIMP-1 knockdown were constructed using si-TIMP-1(1). Additionally, the mRNA expression of *TIMP-1* in the blank cells and cells transfected with TIMP-1-OE plasmid was 1.01 ± 0.12 and 905.98 ± 24.17, respectively, implying that *TIMP-1* expression in the TIMP-1-OE group was approximately 900 times higher than that in the blank group (Figure 1d). TIMP-1 protein and mRNA expression in the blank cells and cells transfected with TIMP-1-OE plasmid was similar (Figure 2e). All the results showed that cells with TIMP-1 overexpression and knockdown were successfully established and could be used for subsequent experiments.

### 3.2 Effects of LC and TIMP-1 on viability of AngII-induced H9C2 cells

**Figure 3:**
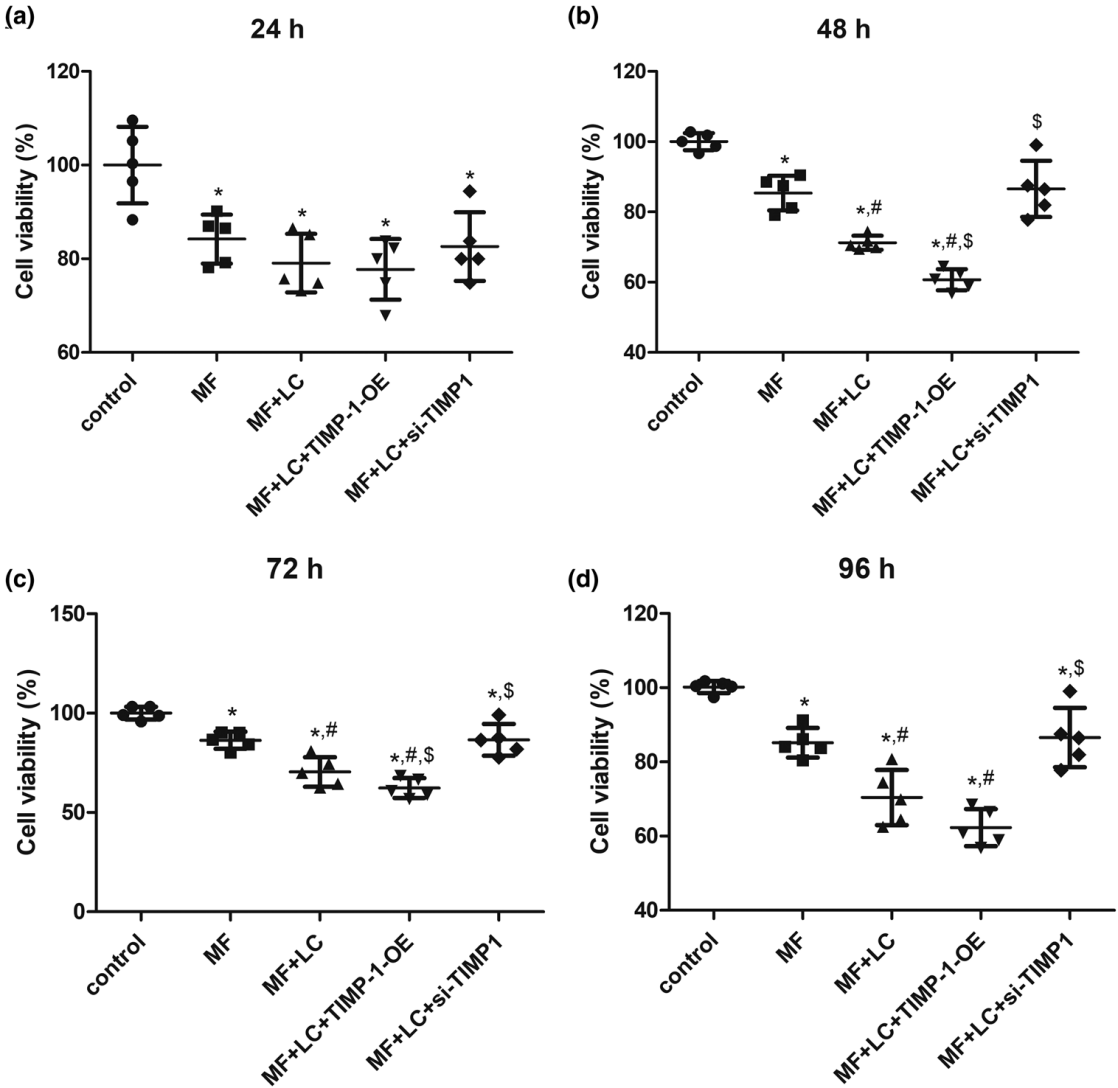
Cell viability of H9C2 cells after si-TIMP-1 and TIMP-1-OE transfection for 24 h (a), 48 h (b), 72 h (c), and 96 h (d). *P < 0.05 vs control, ^#^ *P* < 0.05 vs MF, ^$^ *P* < 0.05 vs MF + LC. TIMP‐1, tissue inhibitor of metalloproteinases‐1; MF, myocardial fibrosis; siRNA, small interfering RNA; OE, over-expression; LC, levocarnitine.

**Figure A1:**
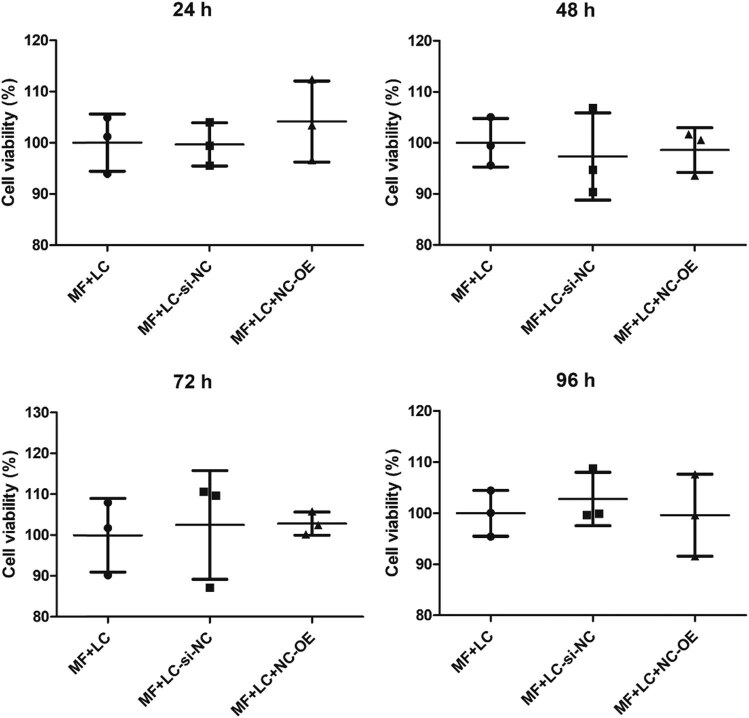
Effects of empty plasmids and transfection reagents on the cell viability of H9C2 cells after culturing for 24, 48, 72, and 96 h using CCK-8.

The viability of H9C2 cells with different treatments for 24, 48, 72, and 96 h was measured using CCK-8. There were no significant differences in cell viability among the MF + LC, MF + LC + si-NC, and MF + LC + NC-OE groups (*P* > 0.05, Figure A1) after culturing for 24, 48, 72, and 96 h, implying that empty plasmids and transfection reagents did not influence cell growth. In addition, AngII treatment for 24, 48, 72, and 96 h significantly inhibited the cell viability relative to the control cells (*P* < 0.05, Figure 3). After treatment for 24 h, no significant difference in cell viability was observed among the MF, MF + LC, MF + LC + TIMP-1-OE, and MF + LC + si-TIMP-1 groups (*P* > 0.05, Figure 3a). After 48, 72, and 96 h of treatment, cell viability was further significantly suppressed by LC compared with that of the MF group (*P* < 0.05) and further decreased after TIMP-1 overexpression compared with that of the MF + LC group (Figure 3b–d). However, compared with that of the MF + LC group, TIMP-1 knockdown increased the viability of AngII-treated cells treated with LC (*P* < 0.05) and restored it to a level similar to that of the MF group (*P* > 0.05, Figure 3b–d). The results suggested that TIMP-1 overexpression could further inhibit the viability of MF cells treated with LC, whereas TIMP-1 knockdown had the opposite effect. Cells cultured for 48 h were used for further experiments.

### 3.3 Effects of LC and TIMP-1 on apoptosis and migration of AngII-induced H9C2 cells

**Figure 4:**
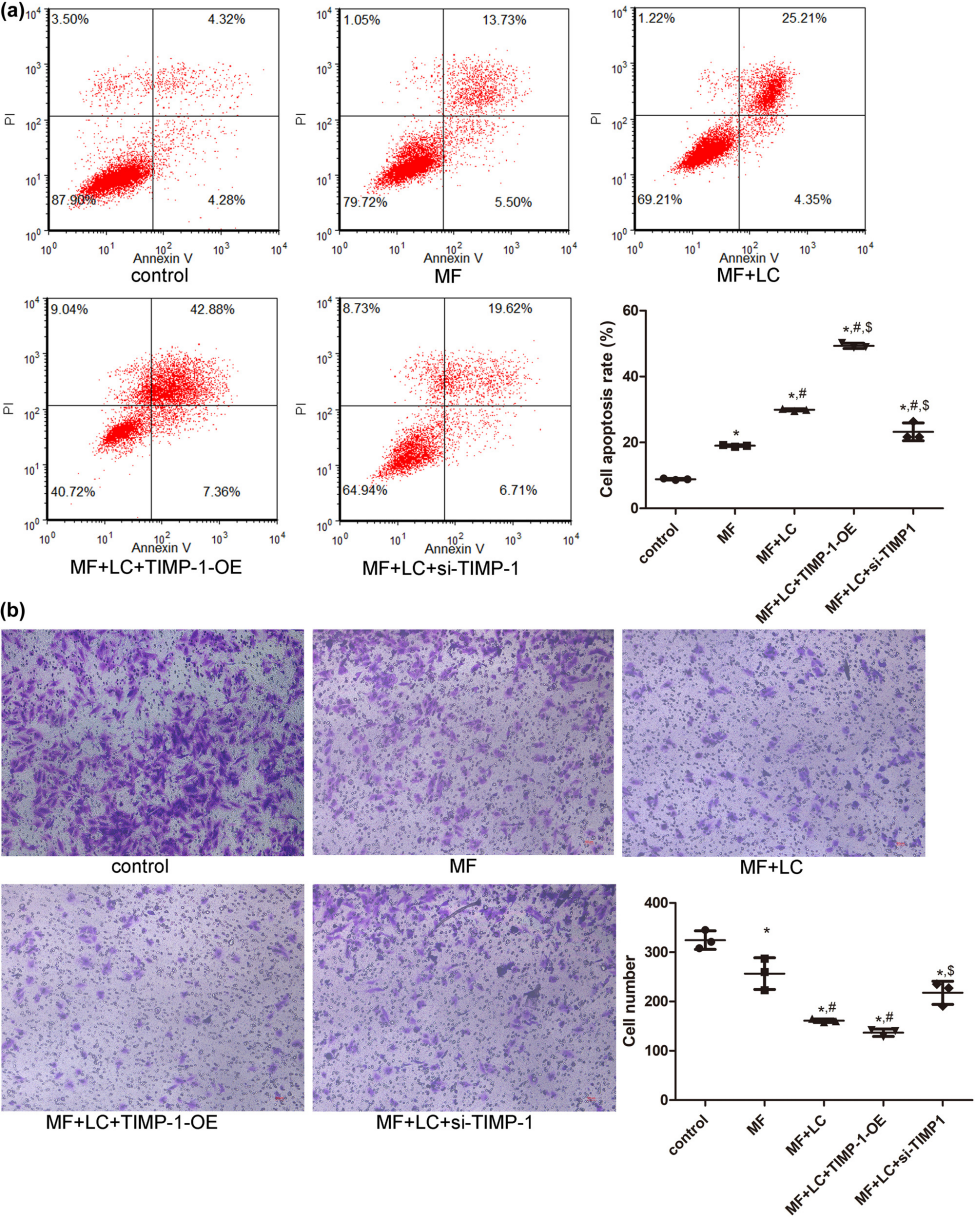
Effects of TIMP-1 on cell apoptosis and migration of H9C2 cells induced by AngII. (a) Cell apoptosis in the different groups determined using flow cytometry. (b) Cell migration in the different groups assessed using tanswell assay. *P < 0.05 vs control, ^#^ *P* < 0.05 vs MF, ^$^ *P* < 0.05 vs MF + LC. TIMP‐1, tissue inhibitor of metalloproteinases‐1; MF, myocardial fibrosis; siRNA, small interfering RNA; OE, over-expression; LC, levocarnitine.

The effects of LC and TIMP-1 on the apoptosis of Ang II-induced H9C2 cells were analyzed using flow cytometry. Compared with that in the control group, a significantly increased rate of cell apoptosis was observed in the MF group (*P* < 0.05, Figure 4a). LC significantly enhanced the apoptosis rate of AngII-induced H9C2 cells compared with the MF group (*P* < 0.05, Figure 4a). Compared with that of the MF + LC group, TIMP-1 overexpression also markedly increased the cell apoptosis rate (*P* < 0.05), whereas TIMP-1 knockdown reduced the apoptosis rate of AngII-induced cells treated with LC (*P* < 0.05, Figure 4a). These results indicated that TIMP-1 overexpression could further promote the apoptosis of MF cells treated with LC, while TIMP-1 knockdown had the opposite effect.

The migration of H9C2 cells with different treatments was examined using the transwell assay. The cell numbers in the control, MF, MF + LC, MF + LC + TIMP-1-OE, and MF + LC + si-TIMP-1 groups were 324.67 ± 18.77, 256.67 ± 32.13, 161.00 ± 3.61, 136.67 ± 7.57, and 217.67 ± 23.44, respectively (Figure 4b). AngII treatment significantly decreased cell numbers compared to that of the control cells (*P* < 0.05), and LC administration and TIMP-1 overexpression further reduced the number of AngII-induced cells (*P* < 0.05, Figure 4b). However, the number of cells in the MF + LC + si-TIMP-1 group was significantly higher than that in the MF + LC group (*P* < 0.05) and reversed to a similar level in the MF group (*P* > 0.05, Figure 4b). These results implied that TIMP-1 overexpression could further suppress the migration of MF cells with LC treatment, whereas TIMP-1 knockdown had the opposite effect.

### 3.4 Effects of TIMP-1 on the expression of oxidative stress-, MF-, and apoptosis-related markers

**Figure 5:**
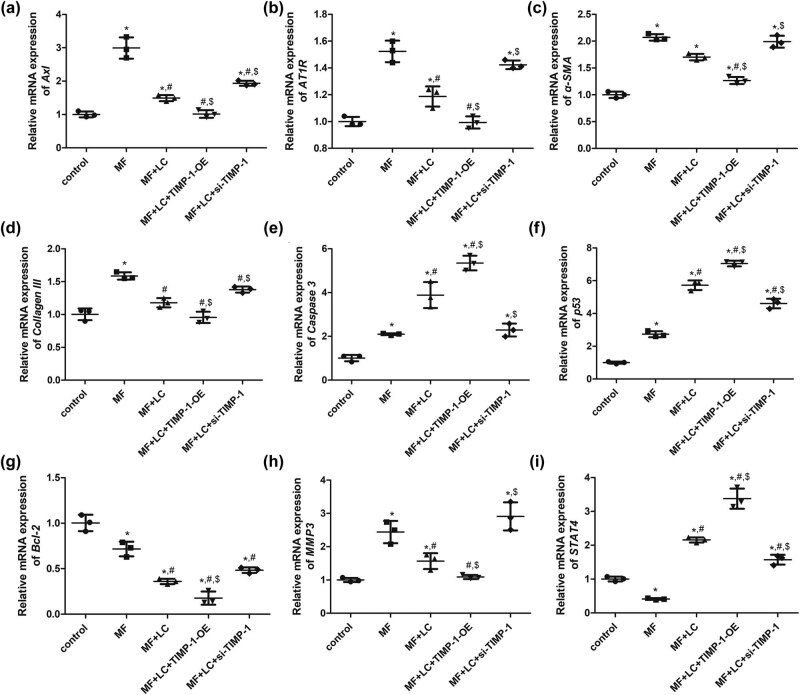
Effects of TIMP-1 on the expression of oxidative stress-related genes, MF-related genes, and apoptosis-related genes determined using qRT-PCR. Relative mRNA expression of *Axl* (a), *AT1R* (b), *α-SMA* (c), *Collagen III* (d), *Caspase 3* (e), *p53* (f), *Bcl2* (g), *MMP3* (h), and *STAT4* (i). *P < 0.05 vs control, ^#^ *P* < 0.05 vs MF, ^$^ *P* < 0.05 vs MF + LC. TIMP‐1, tissue inhibitor of metalloproteinases‐1; MMP, matrix metalloproteinase; Axl, AXL receptor tyrosine kinase; AT1R, angiotensin II receptor type 1; α-SMA, α-smooth muscle actin; MF, myocardial fibrosis; siRNA, small interfering RNA; OE, over-expression, LC, levocarnitine.

To further understand the molecular mechanisms by which TIMP-1 regulates the growth of MF cells, the expression of oxidative stress-related genes (*MMP3* and *STAT4*), MF-related genes/proteins (TIMP-1, Axl, AT1R, α-SMA, and collagen III), and apoptosis-related genes (p53, caspase 3, and Bcl-2) was determined using qRT-PCR and western blotting. qRT-PCR showed that the mRNA expression levels of MF-related genes (*Axl*, *AT1R*, *α-SMA*, and *collagen III*) were significantly upregulated in the MF cells compared with that in the control cells (*P* < 0.05), whereas they were downregulated by LC treatment compared with that in the MF group (*P* < 0.05, Figure 5a–d). Compared with that in the MF + LC group, TIMP-1 overexpression significantly downregulated *Axl*, *AT1R*, *α-SMA*, and *collagen III* mRNA expression (*P* < 0.05), but TIMP-1 knockdown markedly upregulated their expression (*P* < 0.05, Figure 5a–d). For apoptosis-related genes (*p53*, *caspase 3*, and *Bcl-2*), it was found that AngII treatment significantly upregulated *p53* and *caspase 3* expression levels and downregulated that of *Bcl-2* compared to that in the control cells (*P* < 0.05). LC and TIMP-1 overexpression significantly upregulated *p53* and *caspase 3* expression and downregulated that of *Bcl-2* compared with that in the MF cells (*P* < 0.05, Figure 5e–g). However, the effects of TIMP-1 knockdown on *p53*, *caspase 3*, and *Bcl-2* expression were opposite to those of TIMP-1 overexpression (Figure 5e–g). For oxidative stress-related genes (*MMP3* and *STAT4*), *MMP3* expression was significantly higher in the MF group than in the control cells (*P* < 0.05), whereas it was lower in the MF + LC group than in the MF group (*P* < 0.05, Figure 5h). Compared to that in the MF + LC group, TIMP-1 overexpression significantly downregulated *MMP3* (*P* < 0.05), whereas TIMP-1 knockdown significantly upregulated its expression (*P* < 0.05, Figure 5h). The trend of *STAT4* expression in the different groups was opposite to that of *MMP3* expression (Figure 5i).

**Figure 6:**
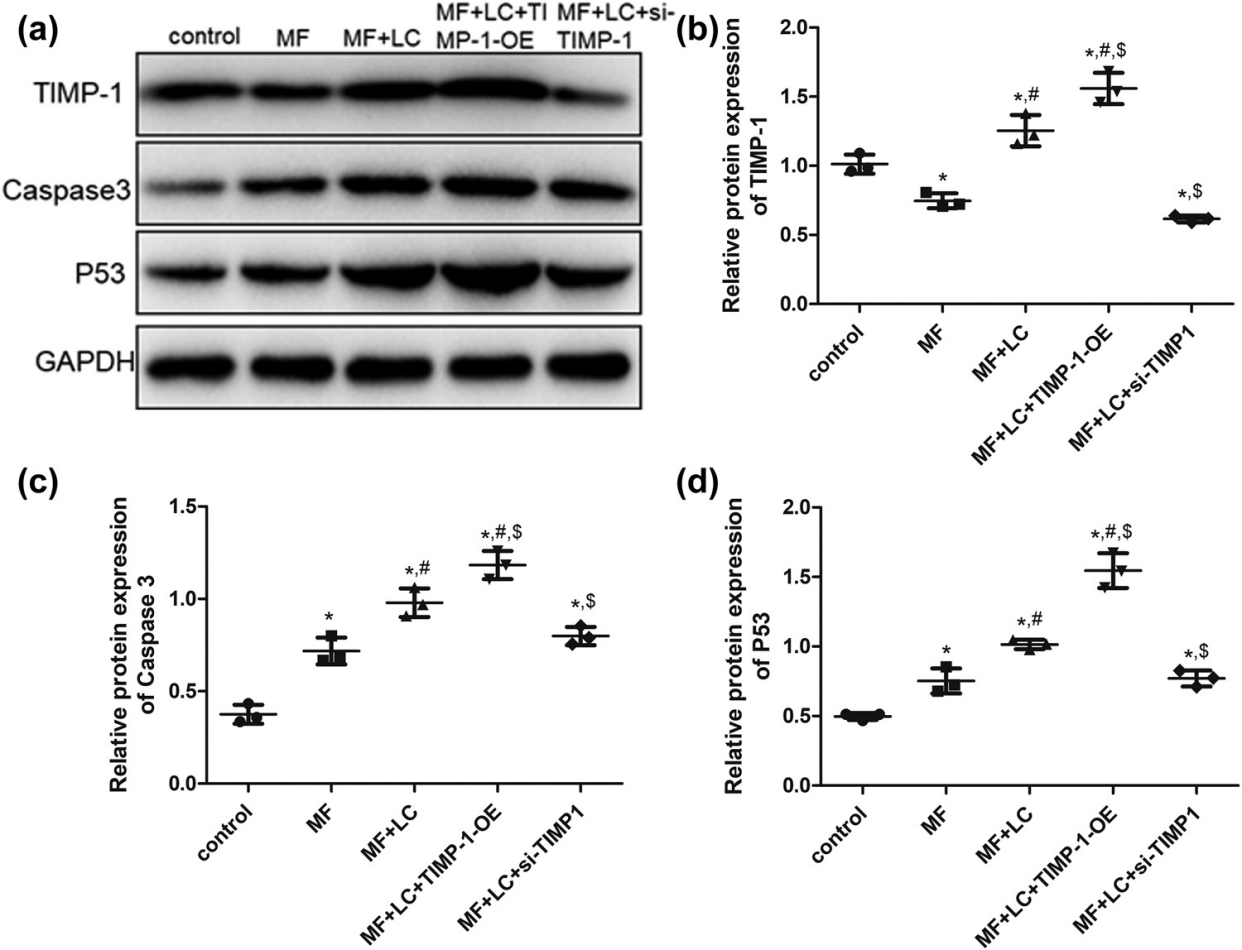
Effects of TIMP-1 on protein expression levels in H9C2 cells determined using western blotting. (a) Representative images of western blotting. The protein expression of TIMP-1 (b), Caspase‑3 (c), and p53 (d). *P < 0.05 vs control, ^#^ *P* < 0.05 vs MF, ^$^ *P* < 0.05 vs MF + LC. TIMP‐1, tissue inhibitor of metalloproteinases‐1; MF, myocardial fibrosis; siRNA, small interfering RNA; OE, over-expression, LC, levocarnitine.

Finally, the protein expression levels of TIMP-1, caspase 3, and p53 were detected using western blotting. The protein expression of TIMP-1 was significantly downregulated after AngII treatment relative to the control cells (*P* < 0.05), while it was upregulated in the MF cells treated with LC (*P* < 0.05, Figure 6a and b). Compared with that in the MF + LC group, TIMP-1 was significantly upregulated by the transfection of TIMP-1 overexpression plasmid (*P* < 0.05), whereas it was downregulated by si-TIMP-1 transfection (*P* < 0.05, Figure 6a and b). Additionally, the amount of caspase 3 and p53 protein expression in the different groups detected using western blotting was similar to that of their mRNA expression examined using RT-qPCR (Figure 6a–d).

## 4 Discussion

In many patients with heart disease, MF is recognized as a common feature, which might ultimately result in organ failure [23]. Extracellular matrix (ECM) protein accumulation within the myocardium is the main characteristic of fibrosis. TIMP-1, upregulated in liver fibrosis development both in murine experimental models and human samples [14], is a significant contributor to greater morbidity and poor prognosis in HF [24]. Therefore, we investigated the role of TIMP-1 in the LC regulation of MF. Lijnen et al. suggested that in rat adult cardiac fibroblasts, a well-established model for fibrosis, Ang II increased collagen secretion and production [25]. In our study, we successfully established an MF cellular model using Ang II, and LC was used to treat Ang II-induced cells. TIMP-1 overexpression and knockdown cells were successfully constructed. AngII induction could inhibit the viability and migration of H9C2 myocardial cells while facilitating their apoptosis, and LC could further suppress the viability and migration of fibrotic myocardial cells and further induce cell apoptosis caused by AngII. qRT-PCR results showed that Ang II induction significantly upregulated the expression of MF-related genes (*Axl*, *AT1R*, *α-SMA*, and *collagen III*), *MMP3*, *caspase 3*, and *p53* and downregulated that of *STAT4* and *Bcl-2*. However, LC reversed the expression of MF-related genes *MMP3* and *STAT4* caused by AngII, further upregulating that of *caspase 3* and *p53* and downregulating that of *Bcl-2*. TIMP-1 overexpression promoted the effects of LC, whereas TIMP-1 knockdown reversed the effects of LC. In light of these findings, TIMP-1 may be a potential therapeutic target for delaying MF progression.

Ang II, a member of the RAS, has been implicated in the development of MF and other fibrotic diseases [26]. A previous study reported that the death of rat myocardial cells triggers an inflammatory response that eventually leads to fibroblast activation and the replacement of dead myocardial cells with fibrous tissue [27]. Wan et al. demonstrated that Ang II treatment induced apoptosis in H9C2 cells, and caspase 3 activity was significantly increased in AngII-induced cells [28]. Our study showed that in MF cells, the viability and migration of myocardial cells were suppressed, whereas their apoptosis was increased. LC is a naturally occurring co-factor involved in fatty acid metabolism, the major biogenesis process in the heart. Therefore, the key role of LC in the pathogenesis of various cardiovascular diseases has been demonstrated [29]. Furthermore, the protective effect of LC on mitochondrial dysfunction and apoptosis has been reported previously [30]. Our study showed that LC could further inhibit the viability and migration of AngII-induced cells while facilitating their apoptosis, in accordance with our previous report. TIMP-1 was upregulated during liver fibrosis and is thought to promote fibrosis in the damaged liver by inhibiting MMP and ECM degradation [31]. However, our study found that TIMP-1 was significantly downregulated in MF cells, which was different from the results reported by a previous study [31]. Therefore, we investigated the role of TIMP-1 in the regulation of MF by LC. Previous evidence also supports the role of TIMPs in various biological processes such as cell differentiation, growth, and apoptosis [32]. Based on the present study, TIMP-1 overexpression could promote the effects of LC, while silencing TIMP-1 could reverse the actions of LC in AngII-induced myocardial cells. Taken together, we speculate that LC combined with TIMP-1 overexpression may be involved in MF progression by further inhibiting the viability and migration of AngII-induced cells while promoting their apoptosis.

To explore the molecular mechanisms of TIMP-1 in the regulation of MF, the expression levels of some molecules associated with oxidative stress (MMP3 and STAT4), apoptosis (Bcl-2, caspase-3, and p53), and MF (TIMP-1, Axl, AT1R, α-SMA, and collagen III) were measured. Our results showed that Ang II exposure significantly upregulated the expression of *Axl, AT1R, α-SMA, collagen III*, *caspase 3*, *p53*, and *MMP3* and downregulated that of *Bcl-2* and *STAT4.* LC treatment reversed the action of AngII on the expression of *Axl, AT1R, α-SMA, collagen III*, *MMP3*, and *STAT4*, whereas it further upregulated that of *caspase 3* and *p53* and downregulated that of *Bcl-2*. TIMP-1 overexpression promoted the effects of LC on these genes, whereas TIMP-1 knockdown had the opposite effect. *Axl*, a receptor tyrosine kinase associated with fibrotic pathways, is involved in myofibroblast activation [33]. *AT1R* can interact with AngII to promote tissue fibrosis and is upregulated in renal fibrosis [34]. *α-SMA* and *collagen III* are fibrosis-related markers that are both upregulated in the fibrosis process. Li et al. [35] illustrated that tetrahydrocurcumin treatment could alleviate diabetic cardiomyopathy by reducing the expression of α-SMA, collagen I, and collagen III, which are markers of cardiac fibrosis.

Oxidative stress is defined as an imbalance between the production of ROS and the endogenous antioxidant defense system. It has been reported to participate in fibrosis and has been identified as an important pathophysiological pathway for the development and progression of HF [36]. Excessive ROS may exacerbate inflammation and progression of HF [37]. ROS can contribute to ECM remodeling by mediating apoptosis and MMPs. *MMP3*, a member of the MMP family, can induce epithelial–mesenchymal transition associated with malignant transformation through a pathway dependent on ROS production [38]. Skacelova et al. [39] observed that MMP3 is highly expressed in patients with rheumatoid arthritis and could serve as a marker of disease activity in rheumatoid arthritis. *STAT4* plays an important role in many diseases through the activation of different cytokines via the JAK-STAT signaling pathway [40]. STAT4 absence could reduce the development of atherosclerosis by affecting MΦ activation and CCL2-induced MΦ migration [41]. Additionally, apoptosis is closely associated with fibrosis [42]. *Caspase‑3*, a member of the caspase family, is a key regulator of extrinsic and intrinsic apoptotic pathways and cell apoptosis [43]. *p53* is an essential cell cycle and DNA repair regulator that plays a role in apoptosis-related genes [44]. *Bcl-2*, an anti-apoptotic gene, is a key downstream effector of the PI3K-Akt signaling pathway that maintains myocardial cell survival and is involved in sudden cardiac death and HF [45]. Combined with our results, it can be inferred that LC together with TIMP-1 overexpression may delay MF by downregulating the expression of MF-related genes (*Axl, AT1R, α-SMA,* and *collagen III*), inhibiting oxidative stress (*MMP3* and *STAT4*), and promoting cell apoptosis (*Bcl2*, *caspase 3*, and *p53*).

However, this study has some limitations. First, TIMP-1 levels should be further determined in clinical samples of patients with MF and paired adjacent benign tissues, and additional investigation into the roles of TIMP-1 in MF should be undertaken *in vivo*. Additionally, the relationship between MF and the growth of Ang II-induced myocardial cells needs to be explored in the future.

## 5 Conclusion

In conclusion, our findings support the hypothesis that LC combined with TIMP-1 overexpression may affect fibrotic cell apoptosis, viability, and migration by regulating the expression of MF-, oxidative stress-, and cell apoptosis-related genes, thereby delaying the progression of MF. Our study provides a theoretical foundation for the treatment of MF-based cardiovascular diseases, with TIMP1 as a potential therapeutic target for LC to promote cardiovascular health and prolong life expectancy in older individuals.
